# Numerical Study on the Dynamic Fracture Energy of Concrete Based on a Rate-Dependent Cohesive Model

**DOI:** 10.3390/ma14237421

**Published:** 2021-12-03

**Authors:** Penglin Zhang, Zhijun Wu, Yang Liu, Zhaofei Chu

**Affiliations:** School of Civil Engineering, Wuhan University, Wuhan 430072, China; zhang_penglin@whu.edu.cn (P.Z.); Richardliuy@whu.edu.cn (Y.L.); zhaofeichu@whu.edu.cn (Z.C.)

**Keywords:** concrete material, fracture energy, high loading rate, cohesive model, failure mode

## Abstract

As an important parameter for concrete, fracture energy is difficult to accurately measure in high loading rate tests due to the limitations of experimental devices and methods. Therefore, the utilization of numerical methods to study the dynamic fracture energy of concrete is a simple and promising choice. This paper presents a numerical investigation on the influence of loading rate on concrete fracture energy and cracking behaviors. A novel rate-dependent cohesive model, which was programmed as a user subroutine in the commercial explicit finite element solver LS-DYNA, is first proposed. After conducting mesh sensitivity analysis, the proposed model is calibrated against representative experimental data. Then, the underlying mechanisms of the increase in fracture energy due to a high strain rate are determined. The results illustrate that the higher fracture energy during dynamic tension loading is caused by the wider region of the damage zone and the increase in real fracture energy. As the loading rate increases, the wider region of the damage zone plays a leading role in increasing fracture energy. In addition, as the strain rate increases, the number of microcracks whose fracture mode is mixed mode increases, which has an obvious effect on the change in real fracture energy.

## 1. Introduction

Concrete is one of the most widely used building materials. Generally, concrete materials are mainly subjected to static loads in a structure, but under certain special conditions, such as explosions and earthquakes, concrete can be subjected to dynamic loads. These special circumstances usually cause huge economic losses and casualties, and therefore, it is important to study the mechanical behavior of concrete under dynamic load. Dynamic strength and fracture energy are the most important parameters that define the onset of fracture in concrete. To date, the former parameter has attracted extensive attention by scholars in attempts to reveal the rate-dependency effect caused by high strain rate loading, whereas the latter parameter has seldom been the focus of research with respect to revealing its rate-dependency effect. Therefore, it is necessary to study the fracture energy of concrete under dynamic loading.

In recent decades, extensive experimental efforts have revealed that both the strength and fracture energy of concrete are quite rate sensitive [1,2,3,4,5,6,7,8,9,10,11,12]. Test methods to study the dynamic fracture energy of concrete include the direct tension test, the split test, the bending test, and the spalling test. Yan and Lin [1] conducted a series of direct tension tests under a relatively low strain rate range (10^−5^–10^−0.3^ s^−1^) using a hydraulic compression device and concluded that fracture is forced to propagate through a region with greater resistance upon a higher strain rate, hence resulting in an increase in fracture energy. Cadoni et al. [2] used a modified Split Hopkinson Bar (SHB) to carry out direct tension experiments on concrete under different strain rates (the largest strain rate being 10 s^−1^). It was determined that the dynamic tensile strength of concrete, ultimate strain, elastic modulus, and fracture energy all increase with increases in strain rate. However, one of the biggest limitations of the direct tension test is how to properly attach the specimen to the testing machine [13]. In addition, the direct tension test is limited to lower strain rates of deformation [14,15,16], up to approximately 10 s^−1^. The split test as an indirect method is also used to test tensile properties [6,7,8]. However, dynamic fracture energy cannot be easily obtained by the split test. Alternatively, bending tests were applied to easily measure fracture energy in previous studies [10,11,12,17,18]. Wittmann et al. [12] conducted three-point bending tests on concrete at different loading rates, and the results showed that fracture energy tends to decrease with increases in loading rate at lower loading rates, but it increases when the loading rate is increased at higher loading rates. Ruiz et al. [11] investigated the influence of the loading rate (1.74 × 10^−5^–1.74 × 10^1^ mm/s) on fracture energy in different high strength concrete and obtained similar results to those of Wittmann et al. [12]. Zhang et al. [10] measured fracture energy and peak load over a wide range of loading rates (1 × 10^−4^–1 × 10^3^ mm/s), and the results showed that they both increase as loading rate increases. Notably, the bending test can only function to a certain low loading rate where the inertia effect is small and inappreciable; however, it cannot be applied at higher loading rates because of larger inertia effect [19]. In order to reach higher levels of strain rates, the spalling technique that utilizes only the input bar of the SHB device is commonly employed [3,4,9,20]. In this technique, one end of the sample is connected to the input bar, and another end is free. Compressive pulse is generated at one end of the input bar via bullet impact on the input bar. When the compressive pulse travels through the bar and arrives at the free end of sample, the tensile pulse is introduced indirectly from the reflection. Brara and Klepaczko [3] applied the spalling technique and reported the effect of high strain rates (higher than 120 s^−1^) in tension on fracture energy. Schuler et al. [4] also applied the spalling technique and measured particle velocity on the free end of the concrete specimen, thus deducing the dynamic strength and fracture energy of the concrete cylinder. It has been embraced that for medium strain rates, a slight moderate increase can be observed until the threshold of approximately 10 s^−1^ is reached, and at such a strain rate, an abrupt increase in fracture energy can be noticed. Although this method can test the tensile properties of concrete at high strain rates, strong assumptions on the material behaviors are introduced to facilitate data processing [21], and it is not clear whether the incident compressive wave will affect the material before the tensile wave returns [13]. Based on the abovementioned studies, researchers have reached a consensus that the fracture energy of concrete increases with increases in loading rate at high loading rates, but a controversy regarding the change trends of concrete fracture energy at low loading rates still exists.

Although all the experimental studies on concrete fracture energy show that there is a definite correlation between loading rate and fracture energy, due to the limitations of the experimental techniques available as well as the difficulty of achieving accurate measurements, experimental data are very scattered. Against this backdrop, numerical simulation provides a powerful means to study the relationship between loading rate and fracture energy. The numerical methods, mainly including (1) continuum-based numerical methods, (2) discontinuum-based numerical methods and (3) hybrid methods, are usually used to study response of concrete under high loading rate. The finite element method (FEM) is a representative continuum-based method, and there are a lot of studies on the dynamic fracture of concrete using this method [5,22,23,24]. FEM usually models dynamic fracturing through damage degradation. However, large element deformation will be generated using the FEM code for a high loading rate simulation, which may lead to difficulties in achieving calculation convergence and to a high cost of computation time. Advanced methods based on FEM have been developed to conquer the weaknesses of FEMs. Extended finite element method (XFEM) adopts an appropriate enrichment function and level set algorithm, thus explicitly representing jumps or discontinuities across the fracture surface and successfully tracing the fracture evolution trajectory, which has managed to be applied to crack propagation of concrete under high loading rate [25,26,27]. However, for an extreme breaking failure, the level set description for the complicated crack patterns is still not robust enough. The discrete element method (DEM), which regards the material as bonded discrete blocks and easily models the material failure process through breaking the bond between two distinct elements and successfully simulating the fragment movement and interaction, is a typical discontinuous-based method. By defining appropriate failure criteria, the method can correctly reflect the overall energy variation, resulting in successful application to modeling the concrete damage and fragmentation process under high loading rate. By defining appropriate failure criteria, the method can correctly reflect the overall energy variation, resulting in successful application to modeling the concrete damage and fragmentation process under high loading rate [28,29]. However, the DEM is stopped from being widely used in large-scale computations due to the large computational cost. Compared with continuum- and discontinuum-based methods, the hybrid method has more advantages in the simulation on dynamic concrete fracture, and it is also a common method in the study of concrete fractures [30,31,32,33]. This method adds cohesive interface elements to the finite element frame to track the initiation and propagation of multiple cracks. The failure of cohesive interface elements can simulate the branching and coalescence of fracture networks and finally form a new free surface. The mechanical properties of the cohesive interface element are described by the constitutive law of rate-dependent cohesive, usually represented by a softening curve. This softening curve represents the gradual loss of strength as the opening degree increases, and the area enclosed by the curve and the coordinate axis represents the dissipated energy. This feature has certain advantages in the study of fracture energy.

Today, the intrinsic mechanisms for the dynamic enhancement of concrete strength are basically understood through three different effects [34,35]: the microcrack inertial effect, material viscosity, and the influence of inertia forces, which can significantly change the state of stresses and strains of the material. However, despite extensive research efforts, questions remain regarding the various mechanisms behind fracture energy increase in concrete materials. The mainstream view is that fracture energy increases during high loading rates due to the structure effect, while real fracture energy is not rate-dependent [13,31,34]. Specifically, there are a number of discrete macro- and microcracks whose total surface area is impossible to accurately measure in dynamic experiments. Moreover, as the loading rate increases, the region of the damage zone is wider, and the energy dissipated by damage will also be calculated as energy dissipated by fracture. Inevitably, the total surface area is underestimated and dissipated energy is overestimated, with the consequence that the evaluated fracture energy is overestimated. In other words, the fracture energy obtained from the dynamic experiment is often not the real fracture energy due to the limitations of technology and equipment. In summary, the structural effects mentioned above definitely exist, but it is not clear whether there are other factors that affect the rate dependence of concrete fracture energy. In this paper, a rate-dependent cohesive model is proposed to study the mechanisms for the increase in the fracture energy of concrete under high loading rates. First, the proposed model is calibrated against representative experimental data, and then concrete tension tests under different strain rates are simulated, and fracture energy is calculated. Finally, the reasons for the increase in concrete fracture energy are discussed from three aspects: the influence of rate-dependent material description, the region of the damage zone, and the failure mode of microcracks.

## 2. Constitutive Law of the Rate-Dependent Cohesive Model

**Figure 1:**
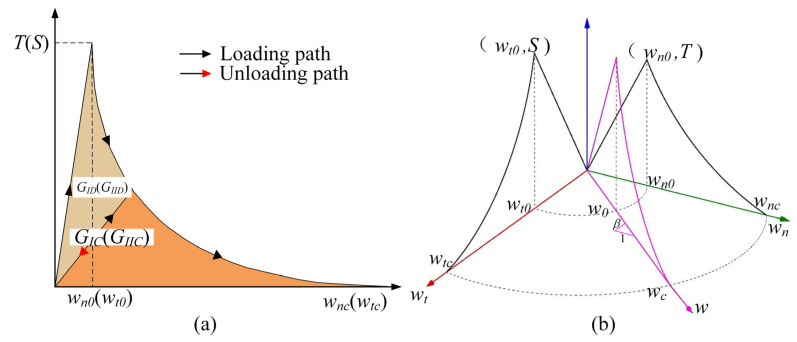
A novel cohesive constitutive model: (**a**) in the pure mode; (**b**) in a mixed model.

In this study, it is assumed that the cracking procedure as well as the nonlinear behavior of concrete are represented by a cohesive constitutive model, and the bulk element adopts a simple linear elastic material model. Although this method is generally consistent with damage evolution in concrete, it is also necessary to select an appropriate constitutive model for cohesive elements to reliably reproduce the damage process [31]. In this regard, cohesive elements were widely used in previous studies to investigate the dynamic fracture of concrete. Though the rate dependency of peak tractions is considered, the rate dependency of the energy release rate is simplified or even ignored in the mode. For example, Zhou et al. [31] assumed that the energy release rate has no rate dependence, and Rosa et al. [36,37] considered that the energy release rate and the peak traction have the same dynamic increase factor (DIF). However, no evidence has been shown that the energy release rate changes in the form of the above two cases. In addition, the curve of the post-peak softening stage is usually simplified as a straight line. From the experimental results [10,18], however, a curve with a different softening rate is closer to the actual situation. A novel rate-dependent cohesive constitutive model is proposed, which has been programmed as a user subroutine in the commercial explicit finite element solver LS-DYNA. The rate dependence of tractions and energy release rate are considered independently as follows:

(1) $$T=T_{0}\times DIF_{T}\;\mathrm{and}\;S=S_{0}\times DIF_{T}$$

(2) $$G_{IC}=G_{IC0}\times DIF_{G}\;\mathrm{and}\;G_{IIC}=G_{IIC0}\times DIF_{G}$$

where *T* and *S* are the peak tractions for pure mode I and pure mode II, respectively. *T*_0_ and *S*_0_ are the peak tractions for pure mode I and pure mode II at the reference loading rate, respectively. Similarly, *G_IC_* is the energy release rate for pure mode I; *G_IIC_* is the energy release rate for pure mode II; *G_IC_*_0_ is the energy release rate for pure mode I at the reference loading rate and *G_IIC_*_0_ is the energy release rate for pure mode II at the reference loading rate. The traction–displacement curve is shown in Figure 1a. *DIF_T_* and *DIF_G_* are the DIFs of peak tractions and energy release rate, respectively, and can be evaluated as:

(3) $$\begin{array}{l} DIF_{T}=1+A\log(\dot{w}/{\dot{w}}_{0}){\left(\dot{w}/{\dot{w}}_{0}\right)}^{M}\\ \mathrm{and}\;DIF_{G}=1+B\log(\dot{w}/{\dot{w}}_{0}){\left(\dot{w}/{\dot{w}}_{0}\right)}^{N} \end{array}$$

where A, B, N, and M are the parameters of the rate-dependent model that need to be calibrated. ${\dot{w}}_{0}$ is the reference opening velocity. $\dot{w}$ is the opening velocity and can be evaluated as:

(4) $$\dot{w}=\sqrt{{\dot{w}}_{n}^{2}+{\dot{w}}_{t}^{2}}$$

where ${\dot{w}}_{n}$ and ${\dot{w}}_{t}$ are the normal opening velocity and tangential opening velocity, respectively. When the displacement is less than the displacement corresponding to the peak traction, the tractions in normal and tangential traction are as follows:

(5) $$f_{t}=\frac{T}{w_{n0}}w_{n}\;\mathrm{and}\;f_{s}=\frac{S}{w_{t0}}w_{t}$$

where *f_t_* and *f_s_* are normal traction and tangential traction, respectively. *w_n_*_0_ and *w_t_*_0_ are the displacement corresponding to normal peak traction and tangential peak traction, respectively. *w_n_* and *w_t_* are normal displacement and tangential displacement, respectively. When the displacement is greater than the displacement corresponding to the peak traction, the softening curve adopts an exponential curve with a different softening rate, and the tractions in normal and tangential traction are as follows:

(6) $$\frac{f_{t}}{T}={\left(1-\sqrt{\frac{w_{n}-w_{n0}}{w_{nc}-w_{n0}}}\right)}^{2}\;\mathrm{and}\;\frac{f_{s}}{S}={\left(1-\sqrt{\frac{w_{t}-w_{t0}}{w_{tc}-w_{t0}}}\right)}^{2}$$

where *w_nc_* and *w_tc_* are the maximum normal displacement and maximum tangential displacement, respectively, and can be evaluated as:

(7) $$\begin{array}{l} w_{nc}=6(G_{IC}-0.5\times T\times w_{n0})/T+w_{n0}\\ \mathrm{and}\;w_{tc}=6(G_{IIC}-0.5\times S\times w_{t0})/S+w_{t0} \end{array}$$

*w_n_*_0_ and *w_t_*_0_ can be evaluated as:

(8) $$w_{n0}=\frac{T_{0}}{K_{0}}\;\mathrm{and}\;w_{t0}=\frac{S_{0}}{K_{0}}$$

where *K*_0_ is the initial stiffness for the cohesive element. The global response of the cohesive model may be affected by this parameter. This parameter be expressed by [31,38]:

(9) $$K_{0}=\frac{\alpha E}{h_{mesh}}$$

where *E* and *h_mesh_* correspond to the Young’s modulus of the bulk material and the element mesh size in the cohesive zone, respectively, and *α* is a constant which needs to be determined. In reference [33], *α* = 50 is recommended. For mixed-mode behavior, the damage initiation displacement is given by:

(10) $$w_{0}=w_{n0}w_{t0}\sqrt{\frac{1+\beta^{2}}{{\left(\beta w_{n0}\right)}^{2}+w_{t0}^{2}}}$$

where *β* is the mode mixity, and *β = w_t_/w_n_*, as shown in Figure 1b. The ultimate displacement for mixed mode is:

(11) $$w_{c}=(1+\beta^{2}){\left[\left(\frac{1}{w_{nc}}\right)+\left(\frac{\beta^{2}}{w_{tc}}\right)\right]}^{-1}$$

In order to better characterize the situation where there is no failure, but damage has occurred in the model, a damage variable is introduced, which can be evaluated as follows:

(12) $$D=\frac{G_{D}}{G_{C}}$$

where *G_D_* is the area enclosed by the unloading path and the previous loading path; *G_C_* is the area enclosed by the loading path. *G_D_* is *G_ID_* and *G_C_* is *G_IC_* when the failure mode is pure mode I, as shown in Figure 1a.

## 3. Model Setup and Calibration

### 3.1. Specimen Geometry and Loading Method

In order to evaluate the reliability of the proposed model for simulating the dynamic fracture of concrete, the test conducted by Cadoni et al. [2] is modeled. The size of the concrete specimen used in the test is a cube with a length of 60 mm. In order to save calculation cost, only two-dimensional simulation is conducted. Therefore, the shape of the model is a square with a length of 60 mm. It should be emphasized that the cohesive constitutive model in LS-DYNA is limited to 3D solid elements, so the plane model is replaced by a model with a layer of 3D solid elements. Therefore, the geometric model for the simulation has a thickness of 1 mm. The lateral boundaries remain stress-free. Displacement constraints are imposed in the out-of-plane direction, such that there is a plane strain state in the structure. Cohesive elements are incorporated between all interfaces between the bulk elements, and the insertion method can refer to our previous research [39].

**Figure 2:**
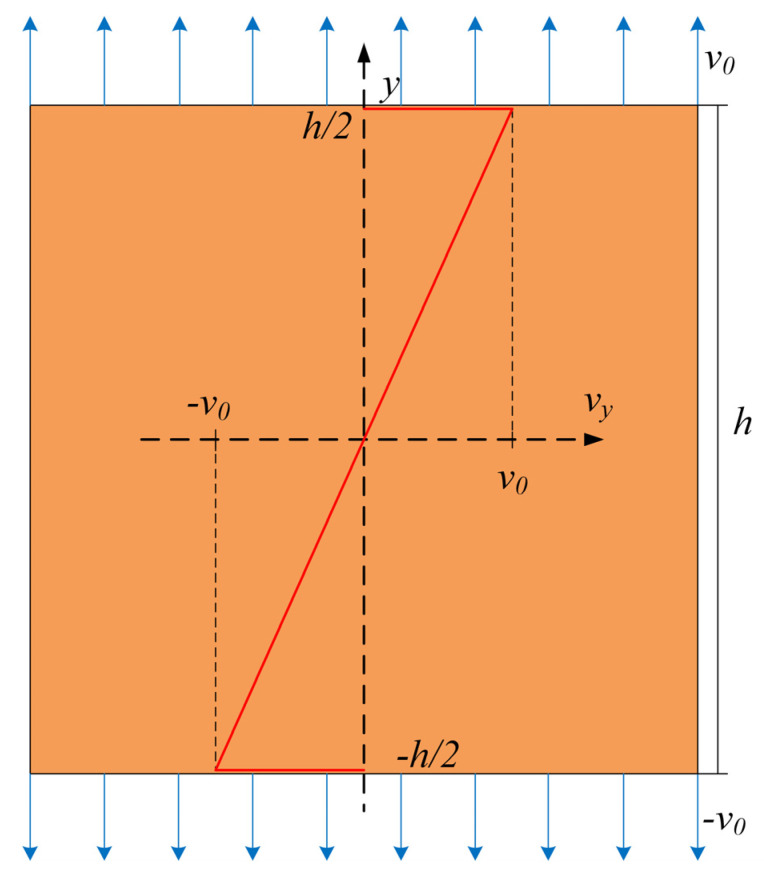
Boundary and initial conditions.

At high loading rates, it is almost impossible to achieve homogeneous stress distribution in concrete specimens. Inhomogeneous stress distribution causes premature failure of the specimen. Therefore, a special loading method is used in this study [13,31,40,41]. As shown in Figure 2, the constant velocity of upper and lower boundaries *v*_0_ can be calculated as follows:

(13) $$v_{0}=\dot{\varepsilon }\frac{h}{2}$$

where $\dot{\varepsilon }$ and *h* correspond to the strain rate and height of the specimen, respectively. The initial velocity of all nodes is calculated by:

(14) $$v(y)=\frac{2v_{0}}{h}y$$

where *y* is the coordinate of the node, as shown in Figure 2.

### 3.2. Model Calibration and Evaluation of DIF

**Figure 3:**
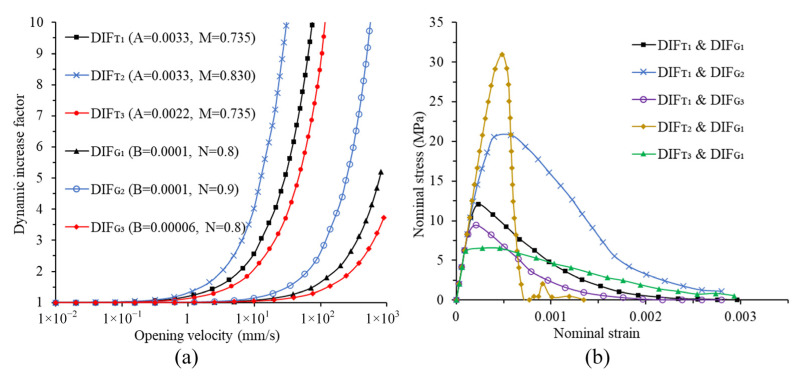
Effect of A, B, M and N on the model and simulation results: (**a**) *DIF_T_* and *DIF_G_* corresponding to different parameters; (**b**) stress–strain curves corresponding to various combinations of *DIF_T_* and *DIF_G_*.

The parameters in the proposed model need to be determined according to experimental data. Firstly, *T*_0_ is basically close to the quasi-static tensile strength obtained from the test, and a tensile strength consistent with the experiment can be obtained after fine-tuning. *G_IC_*_0_ of the model is often smaller than the fracture energy obtained by the quasi-static test, because the energy dissipated by damage is ignored in the test. Generally, after determining *T*_0_, compare the post-peak curves of the tension simulation and the tensile experiment to determine *G_IC_*_0_. *S*_0_ and *G_IIC_*_0_ lack comparable experimental data. The shear properties for the model are set according to the studies in [33], where *S*_0_ and *G_IIC_*_0_ are reported to be 4 times *T*_0_ and 10 times *G_IC_*_0_, respectively. Then, it is necessary to determine the five parameters reflecting the rate dependence. Among them, ${\dot{w}}_{0}$ is the reference opening velocity, selected as 0.01 mm/s, and other parameters need to be calibrated according to experimental data. In Figure 3a, the *DIF_T_* curves corresponding to the three groups of A and M with different values and the *DIF_G_* curves corresponding to the three groups of B and N with different values are drawn. It can be found that when the opening velocity is low, both *DIF_T_* and *DIF_G_* are slowly increasing. As the opening velocity increases to some extent, both *DIF_T_* and *DIF_G_* enter a sharp increase phase. When the opening velocity and A remain the same, the larger M is, the larger *DIF_T_* is; when the opening velocity and M remain the same, the larger A is, the larger *DIF_T_* is. Therefore, both A and M have positive correlation with *DIF_T_*. Similarly, both B and N have positive correlation with *DIF_G_*. In order to further understand the influence of these four parameters on the simulation results, five simulations are conducted with various combinations of *DIF_T_* and DIF_G_ at the strain rate of 10 s^−1^. Figure 3b shows the stress–strain curves corresponding to various combinations of *DIF_T_* and *DIF_G_*. It can be seen from the figure that different combinations of *DIF_T_* and *DIF_G_* mainly affect the peak of stress–strain curve and the slope of the post-peak curve. When adopting the same *DIF_T_* curve and different *DIF_G_* curves, the slope of the post-peak curve does not change significantly, but the *DIF_G_* curve with higher position in Figure 3a can obtain higher peak of stress–strain curve, as shown in the blue, black, and purple curves in Figure 3b. When adopting the same *DIF_G_* curve and different *DIF_T_* curves, the *DIF_T_* curve with higher position in Figure 3a can obtain higher peak of stress–strain curve and larger slope of the post-peak curves, as shown in the yellow, black, and green curves in Figure 3b. Several simulations under different strain rates are then conducted, and by comparing the stress–strain curves obtained by the simulations with the stress–strain curves obtained from the experiment [2], the values of A, B, M and N are calibrated. All parameters after calibration are shown in Table 1.

**Figure 4:**
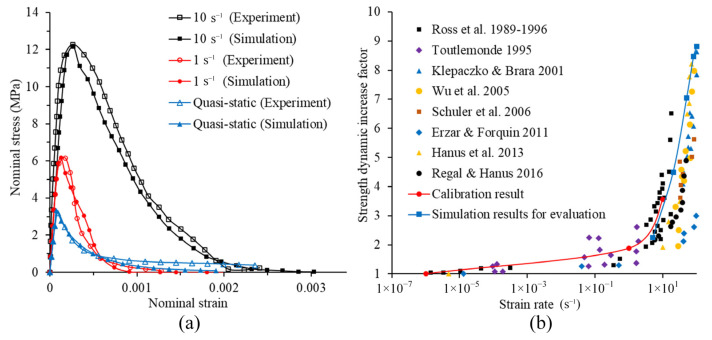
Model calibration and evaluation of DIF with experimental results: (**a**) stress–strain curves of simulations and experiments [2]; (**b**) DIF of tension strength for simulations and experiments [42].

Figure 4 shows the comparison between simulation results using the calibrated parameters and experimental data. The experimental stress–strain curves used for calibration and the stress–strain curves of the simulations using the calibrated parameters are shown in Figure 4a. It can be seen from the figure that the simulation results have good overall consistency with the experimental data, indicating that the parameter calibration is successful. Furthermore, in order to evaluate the reliability of the proposed model, the simulations using the calibrated parameters with strain rates of 5 s^−1^, 20 s^−1^, 50 s^−1^, 80 s^−1^ and 100 s are conducted. As can be seen from Figure 4b, the DIF predicted by the model and the experimental DIF have a reasonable correlation under different strain rates, which proves that the model is capable to predict the response of concrete under high tensile loading rate. Thus, the model is further applied to analyze the intrinsic mechanisms which govern the increase in dynamic fracture energy.

### 3.3. Mesh Sensitivity Analysis

**Figure 5:**
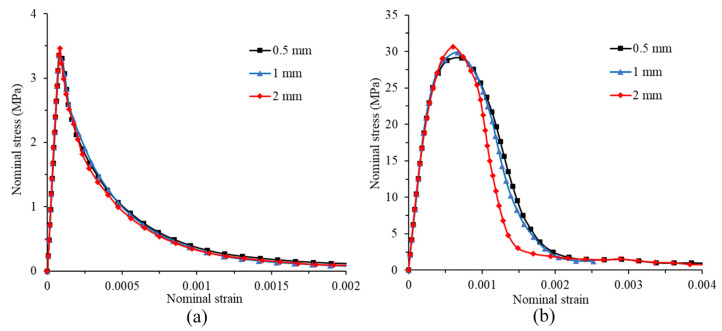
Stress–strain curve under different mesh sizes: (**a**) quasi-static loading; (**b**) $\dot{\varepsilon }=$ 100 s^−1^.

In order to analyze the mesh sensitivity of the model, the tension simulations of the model with three mesh sizes (0.5 mm, 1 mm and 2 mm) under two different tensile strain rates (1 × 10^−6^ s^−1^ and 100 s^−1^) are conducted. Figure 5 shows the nominal stress–strain curve under two different strain rates. The nominal stress is calculated as the boundary loading force divided by the cross section area. The nominal strain is determined by the sum of the upper and lower boundaries divided by the height of the specimen. The nominal strength refers to the peak value of nominal stress. From Figure 5a, it can be found that the stress–strain curves obtained by the simulation of different mesh sizes in quasi-static state almost coincide. This shows that the effect of mesh size is almost negligible at low strain rates. However, when strain rate is 100 s^−1^, the stress–strain curve obtained with a mesh size of 2 mm obviously deviates from other curves, especially after the curve peak, as shown in Figure 5b. However, the curve with a mesh size of 1 mm is very close to the curve with a mesh size of 0.5 mm, which means that using a mesh size of 1 mm appears to be good enough even for a high strain rate. Therefore, in subsequent simulations, a mesh size of 1 mm is used.

## 4. Numerical Investigation on Dynamic Fracture Energy

### 4.1. Damage and Unpenetrated Crack

**Figure 6:**
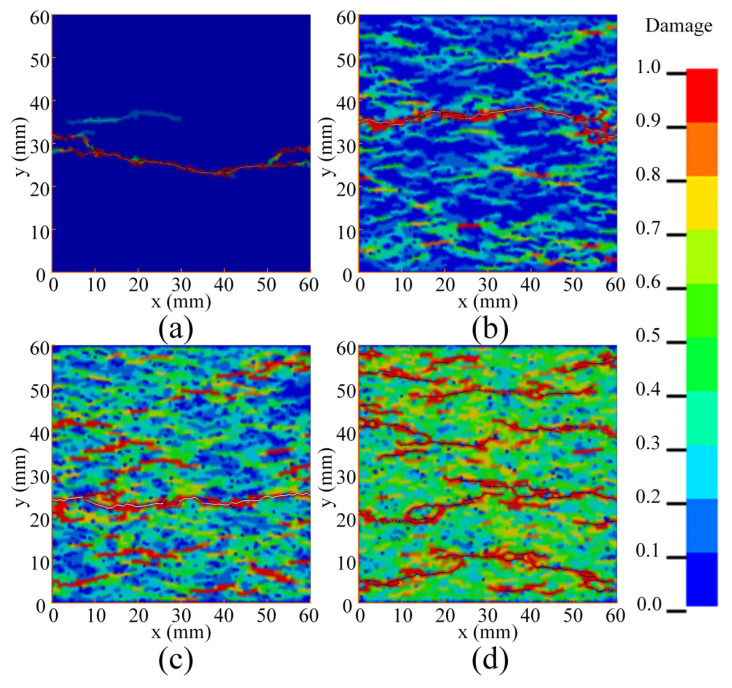
Damage of specimen under different strain rates: (**a**) quasi-static; (**b**) $\dot{\varepsilon }=$ 1 s^−1^; (**c**) $\dot{\varepsilon }=$ 10 s^−1^; (**d**) $\dot{\varepsilon }=$ 100 s^−1^.

Some researchers believe that the increase in concrete fracture energy in the experiment is due to the structural effect [13,31]. In order to study the influence of structural effects on fracture energy, it is necessary to study the damage of concrete specimens under different strain rates. Figure 6 shows the damage of the specimens under different strain rates. It should be pointed out that in Figure 6, the damage value of the cohesive elements is displayed on the adjacent bulk elements for more intuitiveness. In the quasi-static state, there is only one penetrating crack, and there is basically no damage in any other region except in the crack position, as shown in Figure 6a. When the strain rate is 1 s^−1^, there is still only one penetrated crack, but obvious damage and a few unpenetrated cracks can be observed in the other regions, as shown in Figure 6b. When the strain rate increases to 10 s^−1^, several unpenetrated cracks randomly distribute on the entire specimen and most regions are damaged, though there is only one penetrated crack, as shown in Figure 6c. When the strain rate increases to 100 s^−1^, the whole region is damaged, and there are several penetrated cracks and a large number of unpenetrated cracks, as shown in Figure 6d. It can be found that as the strain rate increases, damaged areas and the number of cracks increase significantly.

**Figure 7:**
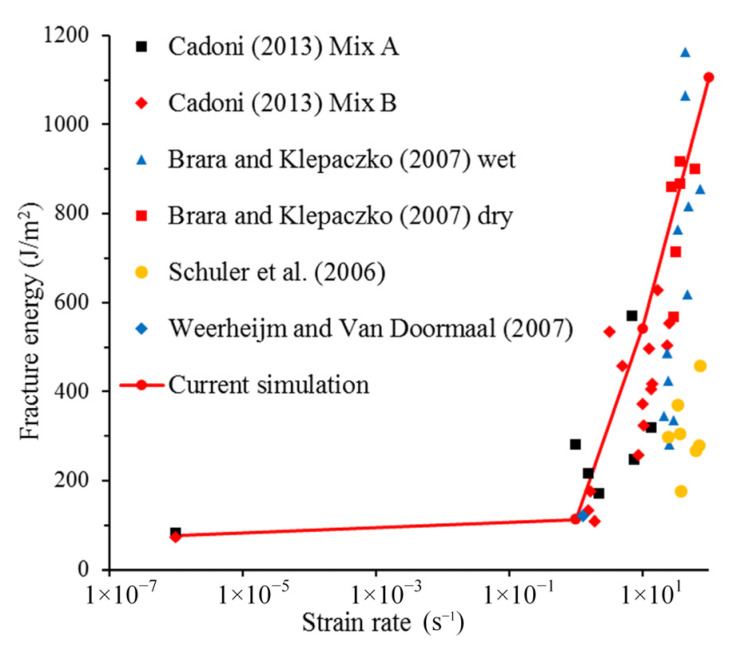
Fracture energy under different strain rates obtained by simulation and experimental results [2].

For the simulation results of concrete dynamic tension, fracture energy can be calculated as follows:

(15) $$G_{f}=(W-\Delta E_{D})/A_{f}$$

where *G_f_* is the fracture energy, W is the work carried out by the boundary loading force, and Δ*E_D_* is the increment in the kinetic energy. In order to avoid premature failure caused by inhomogeneous stress distribution, the model has been specially treated, and the special treatment makes the model have initial kinetic energy. In addition, kinetic energy cannot be completely dissipated at the end of the simulation, such that the increase in kinetic energy needs to be subtracted. A*_f_* is the fracture area. In order to compare with experimental data, only the area of the penetrated cracks is calculated. The calculated fracture energy of concrete at different strain rates is shown in Figure 7. It can be found that the simulation results are in good agreement with the experimental results. This shows that the structural effect does make a great contribution to the increase in fracture energy. Simultaneously, it proves the reliability of this model again.

### 4.2. The Real Fracture Energy

In the previous section, it has been proved that the structural effect makes a significant contribution to the increase in fracture energy. However, it is not clear whether the real energy dissipated per unit area at the fracture location, that is, the real fracture energy, changes with the strain rate. Real fracture energy is closely related to the energy release rate defined in the model. When cohesive element failure occurs at a constant velocity in the normal direction, the *G_IC_* in the model is the real fracture energy. Similarly, when a cohesive element failure occurs at a constant velocity in tangential traction, the *G_IIC_* in the model is the real fracture energy. When mixed failure occurs, the real fracture energy is calculated by *G_IC_* and *G_IIC_*. Therefore, by verifying whether *G_IC_* and *G_IIC_* have rate dependence, it can be proved whether the real fracture energy has rate dependence.

**Figure 8:**
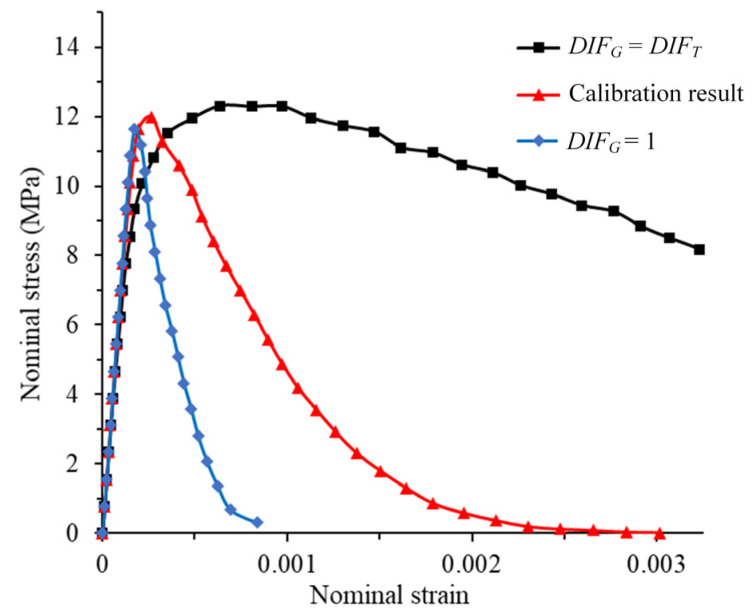
Stress–strain curve for different *DIF_G_*.

**Figure 9:**
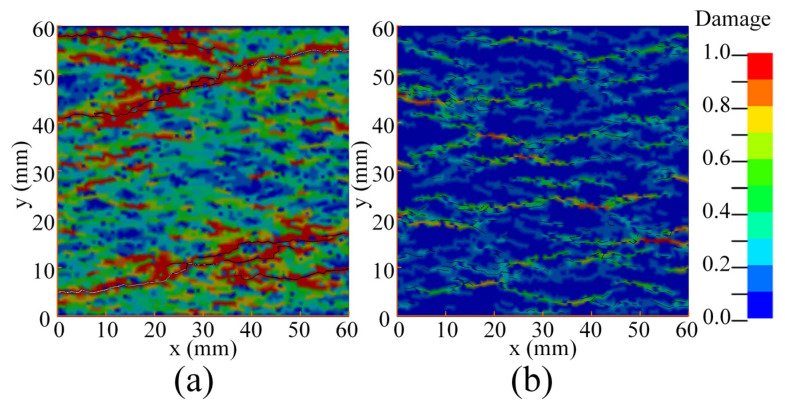
Damage of specimen: (**a**) result of *DIF_G_* = 1; (**b**) result of *DIF_G_* = *DIF_T_*.

A simulation without considering the DIF of the energy release rate was conducted when the strain rate was 10 s^−1^ (*DIF*_G_ = 1). In addition, another simulation was conducted in which the DIF of energy release rate was equal to the DIF of peak traction (*DIF_G_* = *DIF_T_*). This is because in the studies of some researchers, the *DIF_G_* is set to be the *DIF_T_* [36,37]. The stress–strain curve is shown in Figure 8. It can be found that when *DIF_G_* = 1, the pre-peak part of the stress–strain curve is very close to the calibrated result, and the peak value of the stress–strain curve is slightly lower than the calibrated result. However, the post-peak stress is significantly smaller than the calibrated result, and its ultimate strain is much smaller than the calibration result. By contrast, when *DIF_G_* = *DIF_T_*, the peak value of the stress–strain curve is higher than the calibrated result, and the post-peak stress is significantly higher than the calibrated result. The stress–strain curve shows strong plasticity, and strength still remains when the strain reaches 0.003. The corresponding damage is shown in Figure 9. Compared with the calibration result, the simulation result has two penetrated cracks when *DIF_G_* = 1 (as shown in Figure 9a), but there is only one penetrated crack in the calibration result (as shown in Figure 6c). In addition, the damage range is also significantly larger than the calibration result. When *DIF_G_* = *DIF_T_*, part of the specimen is damaged, but no failure has occurred, and the damage range is much smaller than the calibration result, as shown in Figure 9b. Through this evidence, it can be confirmed that the real fracture energy has rate dependence, and this property has a great influence on the break of the concrete specimen.

**Figure 10:**
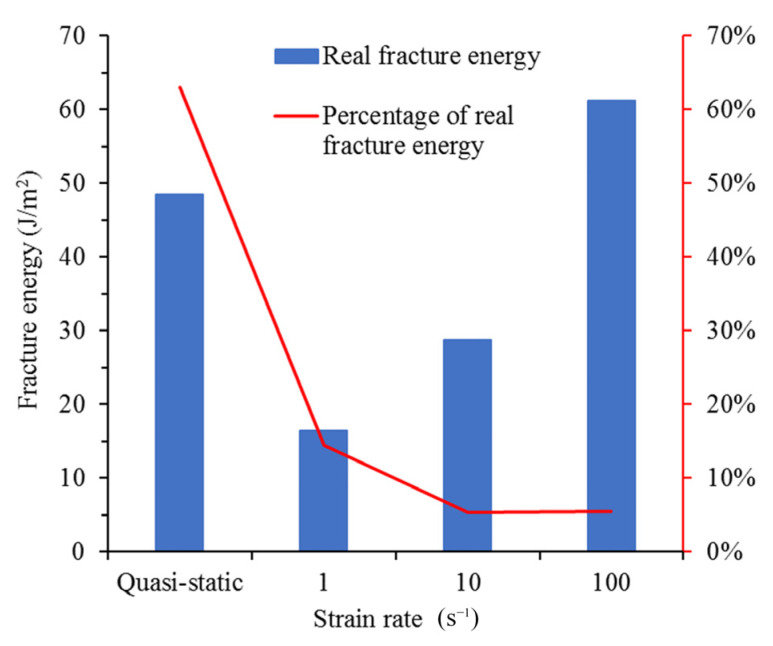
Real fracture energy of concrete under different strain rates.

Although all cracks can be measured in the simulation, the energy dissipated by the damage is still not easy to calculate. Therefore, the method in Section 4.1 cannot be used to calculate the real fracture energy. The only way to obtain the real fracture energy is to record the energy of cohesive elements dissipated at every time step during the simulation process. After the simulation, the sum of the energy dissipated by all failed cohesive elements divided by the sum of the area of all failed cohesive elements is the real fracture energy. The real fracture energy of concrete is shown in Figure 10.

**Figure 11:**
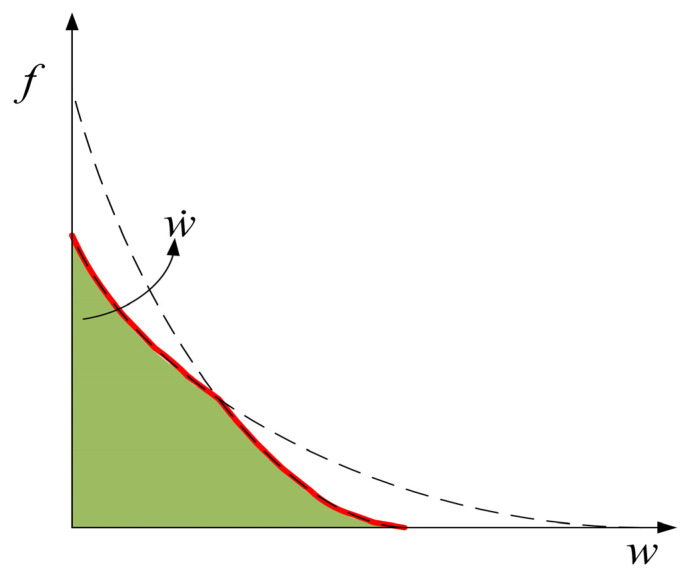
Traction–opening curve under different open velocities.

The real fracture energy of the quasi-static state is greater than when the strain rate is 1 s^−1^. This shows that, within the range of strain rate from 1 × 10^−6^ to 1 s^−1^, there must be an interval where the real fracture energy decreases with an increase in strain rate. Since the structural effect will definitely increase with the increase in strain rate, fracture energy increases very little or even slightly decreases in this interval. Similar results have also been found in some experimental studies [11,12]. The reason this phenomenon occurs in the simulation results is that the peak traction of the cohesive element increases significantly, but the energy release rate does not in this strain rate interval (see Figure 3). It can be seen from Equation (7) that the maximum displacement will decrease in this case. As shown in Figure 11, the opening velocity increases due to the continuous loss of strength, so the true traction-opening curve may be as shown in the red line in Figure 11. The area enclosed by the red line and the coordinate axis is significantly smaller than the area enclosed by the quasi-static curve, in other words, the real fracture energy decreases as the strain rate increases. It can be seen in Figure 11 that when the strain rate increases from 1 to 100 s^−1^, the real fracture energy increases from 18 J/m^2^ to 63 J/m^2^. This illustrates that when the strain rate is greater than 1 s^−1^, fracture energy increases with the increase in strain rate. The red line in Figure 10 represents the proportion of the real fracture energy in the calculated fracture energy (the real fracture energy value is divided by the fracture energy calculated in Figure 6). It can be found that as the strain rate increases, the proportion of the real fracture energy continues to decrease. In the quasi-static state, the proportion reaches 64%. The proportion is reduced to 15% when the strain rate is 1 s^−1^. When the strain rate is greater than or equal to 10 s^−1^, the proportion is only 5%. It is indicated that when the strain rate is low, the real fracture energy has a significant effect on fracture energy. When the strain rate is high, although the real fracture energy increases with the increase in strain rate, the main factor for the increase in fracture energy is the structural effect.

### 4.3. The Failure Mode of Microcracks

Some researchers believe that with the increase in loading rate, the failure mode of microcracks also changes. In general, there is a tendency for the failure mode to change from mode I to mixed mode with the increase in loading rate [43,44]. Through acoustic emission technology, Chen et al. [18] proved that the proportion of shear failure increases with the increase in strain rate. Does the microcrack’s failure mode really change at high loading rates? Is there a certain connection between the change in the failure mode and the rate dependence of fracture energy? This section discusses the above two issues. 

**Figure 12:**
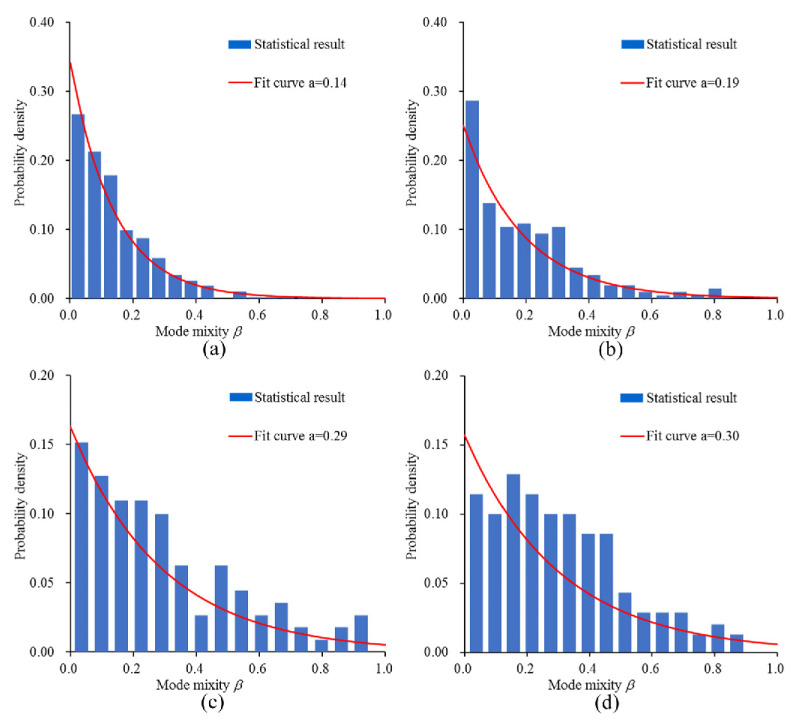
Mode mixity of failed cohesive element statistics under different strain rates: (**a**) quasi-static; (**b**) 1 s^−1^; (**c**) 10 s^−1^; (**d**)100 s^−1^.

In the rate-dependent cohesive model used in this study, parameter *β* can reflect the failure mode of the failed cohesive element (form Equation (10)). From its definition, it can be seen that when *β* is close to zero, it means that the failure mode is close to mode-I failure. When *β* approaches infinity, the failure mode is close to mode-II failure. In the simulations, in order to explain the change in the microcrack’s failure mode, the *β* of all the failed cohesive elements is output. Figure 12 shows the *β* value statistics of all failed cohesive elements under different strain rates. In the figure, a is the average value of *β* of all failed cohesive elements. In the quasi-static state, the *β* value of the failed cohesive elements obeys an exponential distribution, and the average value is 0.14, as shown in Figure 12a. The *β* of most failed cohesive elements is close to 0, which indicates that the failure mode of most cohesive elements is close to pure mode I in the quasi-static state. When the strain rate is 1 s^−1^, the *β* distribution basically obeys the exponential distribution. The *β* of most failed cohesive elements is also close to 0, but compared to the quasi-static state, the average value has increased to 0.19, as shown in Figure 12b. Compared with the strain rate of 1 s^−1^, when the strain rate increases to 10 s^−1^, the number of failed cohesive elements with a *β* greater than 0.6 increases significantly, and the average value increases to 0.29, as shown in Figure 12c. When the strain rate is increased to 100 s^−1^, the number of failed cohesive elements with *β* in the range of 0.2–0.4 is the largest, and the average value also reaches 0.30, as shown in Figure 12d. When the strain rate increases from 10 to 100 s^−1^, the average value of *β* does not increase significantly. From the change in *β*, it can be seen that with the increase in strain rate, the failure mode of the most microcracks changes from mode I to mixed mode. However, when the strain rate is greater than 10 s^−1^, the average value of *β* begins to become constant, which means that as the strain rate increases, the failure mode of most microcracks first changes from close mode I to mixed mode, and then stabilizes with constant mode mixity.

**Figure 13:**
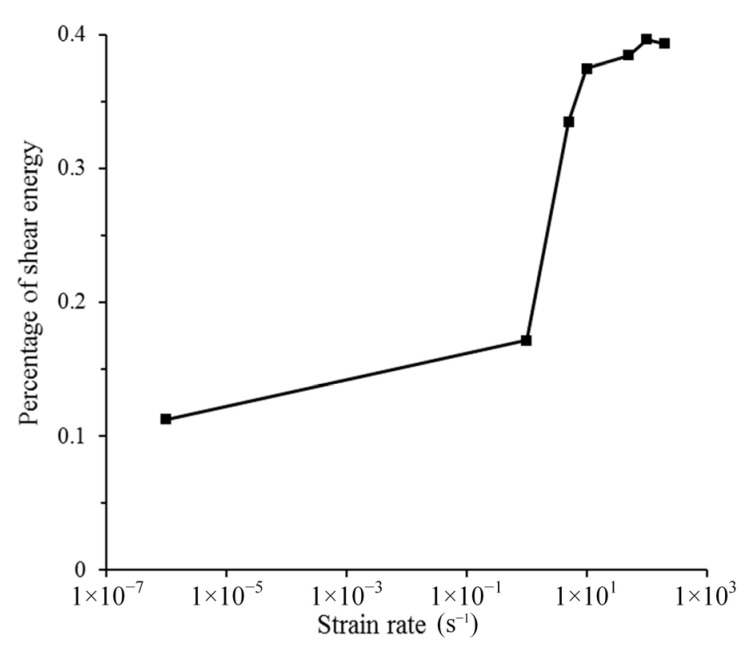
The percentage of shear energy in all dissipated energy.

It has been proven that the failure mode of microcracks will change with the increase in strain rate; specifically, mode mixity will increase with the increase in strain rate. According to the definition of mode mixity, an increase in mode mixity means an increase in the ratio of tangential displacement to normal displacement. Since tangential peak traction and energy release rate are much greater than the normal direction, if the *β* value increases, the energy dissipated by the failed cohesive element will increase, and the real fracture energy will also increase. The real fracture energy is obtained by outputting the dissipated energy and area of all failed cohesive elements. In fact, the dissipated energy of the cohesive element is the sum of the work carried out by normal traction and tangential traction. In order to prove the above point, the percentage of shear energy under different strain rates is calculated (add strain rate of 5 s^−1^, 50 s^−1^, and 200 s^−1^), as shown in Figure 13. It can be found that in the quasi-static state, shear energy is only a small proportion, which shows that the failure mode is closer to the mode-I fracture. As the strain rate increases, the proportion of shear energy continues to increase. When the strain rate is greater than 10 s^−1^, the proportion of shear energy tends to be constant, which is possibly because the *β* of most failed cohesive elements tends to be constant. In summary, the change in the microcrack failure mode has an obvious effect on the increase in real fracture energy.

## 5. Conclusions

In this study, a novel rate-dependent cohesive constitutive was proposed and successfully applied to simulate the dynamic tension test of concrete materials. The softening stage of this constitutive takes an exponential curve, and the rate dependences of peak traction and energy release rate are independently considered. After verification, a direct tensile test of concrete under four different strain rates (quasi-static, 1, 10, and 100 s^−1^) was simulated. Finally, the influence of the structural effect, the rate dependence of the real fracture energy, and the fracture mode of the microcracks on the change in fracture energy with the strain rate were analyzed. Based on the results, the main conclusions can be drawn as follows:

(1) Two reasons contribute to the fact that fracture energy changes when increasing the strain rate. One is that the damage range and unmeasurable cracks of concrete increase with the increase in strain rate (structural effect), and the other is the rate dependence of concrete real fracture energy;
(2) As the strain rate increases, the damage range on the specimen becomes larger, and the number of unpenetrated cracks increases, which leads to more pronounced structural effects;
(3) There is a sub-interval in the range of strain rate from 1 ×10^−6^ to 1 s^−1^, where the real fracture energy decreases with the increase in strain rate. With the strain further increasing to 1 s^−1^, the real fracture energy increases with the increase in strain rate. However, despite the increase in real fracture energy at this stage, the proportion of real fracture energy in fracture energy drops, indicating that structural effects dominate the increase in fracture energy;
(4) As the strain rate increases, more microcracks will change from mode-I type to mixed fracture mode. Meanwhile, change in fracture mode has obvious effects on real fracture energy.

## Figures and Tables

**Table 1:** Parameters for the model.

Bulk Element	Density *ρ* (kg/m^3^)		Young’s Modulus E (GPa)		Poisson’s Ratio υ
	2418		44.9		0.2
cohesive element	*T*_0_ (MPa)	*S*_0_ (MPa)	*G*_*IC*0_ (J/m^2^)	*G*_*IIC*0_ (J/m^2^)	*α*
	3.3	13.2	40	400	50
	A	B	M	N	${\dot{w}}_{0}$ (mm/s)
	0.0033	0.0001	0.735	0.8	0.01
